# Stem cell-based therapy for COVID-19 and ARDS: a systematic review

**DOI:** 10.1038/s41536-021-00181-9

**Published:** 2021-11-08

**Authors:** Gabriele Zanirati, Laura Provenzi, Lucas Lobraico Libermann, Sabrina Comin Bizotto, Isadora Machado Ghilardi, Daniel Rodrigo Marinowic, Ashok K. Shetty, Jaderson Costa Da Costa

**Affiliations:** 1 Brain Institute of Rio Grande do Sul (BraIns), Pontifical Catholic University of Rio Grande do Sul (PUCRS), Porto Alegre, RS Brazil; 2 Graduate Program in Medicine, Pediatrics and Child Health, Pontifical Catholic University of Rio Grande do Sul (PUCRS), Porto Alegre, RS Brazil; 3 School of Medicine, Pontifical Catholic University of Rio Grande do Sul (PUCRS), Porto Alegre, RS Brazil; 4 Institute for Regenerative Medicine, Department of Molecular and Cellular Medicine, Texas A&M University College of Medicine, College Station, TX USA

**Keywords:** Stem-cell therapies, Stem-cell research, Viral infection, Respiratory tract diseases

## Abstract

Despite global efforts to establish effective interventions for coronavirus disease 2019 (COVID-19) and its major complications, such as acute respiratory distress syndrome (ARDS), the treatment remains mainly supportive. Hence, identifying an effective and safe therapy for severe COVID-19 is critical for saving lives. A significant number of cell-based therapies have been through clinical investigation. In this study, we performed a systematic review of clinical studies investigating different types of stem cells as treatments for COVID-19 and ARDS to evaluate the safety and potential efficacy of cell therapy. The literature search was performed using PubMed, Embase, and Scopus. Among the 29 studies, there were eight case reports, five Phase I clinical trials, four pilot studies, two Phase II clinical trials, one cohort, and one case series. Among the clinical studies, 21 studies used cell therapy to treat COVID-19, while eight studies investigated cell therapy as a treatment for ARDS. Most of these (75%) used mesenchymal stem cells (MSCs) to treat COVID-19 and ARDS. Findings from the analyzed articles indicate a positive impact of stem cell therapy on crucial immunological and inflammatory processes that lead to lung injury in COVID-19 and ARDS patients. Additionally, among the studies, there were no reported deaths causally linked to cell therapy. In addition to standard care treatments concerning COVID-19 management, there has been supportive evidence towards adjuvant therapies to reduce mortality rates and improve recovery of care treatment. Therefore, MSCs treatment could be considered a potential candidate for adjuvant therapy in moderate-to-severe COVID-19 cases and compassionate use.

## Introduction

In January 2020, to raise awareness internationally and to prevent further viral spread, the World Health Organization (WHO) declared the severe acute respiratory syndrome coronavirus 2 (SARS-CoV-2) outbreak a public health emergency^1^. The virus responsible for coronavirus disease 2019 (COVID-19) infected more than 128.7 million people around the world by March 2021^2^. Despite global efforts to establish effective interventions for COVID-19, its treatment remains mainly supportive, and one of the major complications of the disease, acute respiratory distress syndrome (ARDS), poses a significant challenge^3^.

Coronaviruses that are responsible for severe respiratory syndrome (SARS) and the Middle East respiratory syndrome (MERS) belong to the same beta coronavirus genus, and SARS and MERS resemble SARS-CoV-2 symptoms^4^. Novel therapeutic approaches have been developed to treat COVID-19 complications, especially ARDS, some of which exploit antiviral and immune-based mechanisms^5^. More recently, a significant number of cell-based therapies have been through clinical investigation, involving, most importantly, mesenchymal stem cells (MSCs) and MSC-derived conditioned media or extracellular vesicles. These therapies have multiple therapeutic targets because MSCs can release a variety of soluble mediators, but their safety and potential efficacy are still to be determined^6^.

Specifically, MSCs secrete keratinocyte growth factor, prostaglandin E2, granulocyte-macrophage colony-stimulating factor, and cytokines such as IL-6 and IL-13, that can influence how immune cells—both innate and adaptive—interact with the cellular environment. These soluble factors can promote alveolar macrophage phagocytosis and alter the cytokine profile released by immune cells. Such functions are expected to be effective against the respiratory infections discussed here^7^.

Considering the limited availability of effective therapies for COVID-19 and one of its complications ARDS, new therapeutic approaches are urgently needed. In this context, cell-based therapies can be an attractive alternative because of their accessibility and relative safety. Although the use of MSCs as immune therapy has been regarded as safe, a meta-analysis found fever as the predominant adverse event associated with MSC therapy. Notably, no acute infusion toxicities, infections, thrombotic/embolic events, or malignancy were found^8^. Here, we systematically reviewed studies that investigated different types of cells as a treatment for the respiratory diseases mentioned above.

## Results

### Study characteristics

**Fig. 1:**
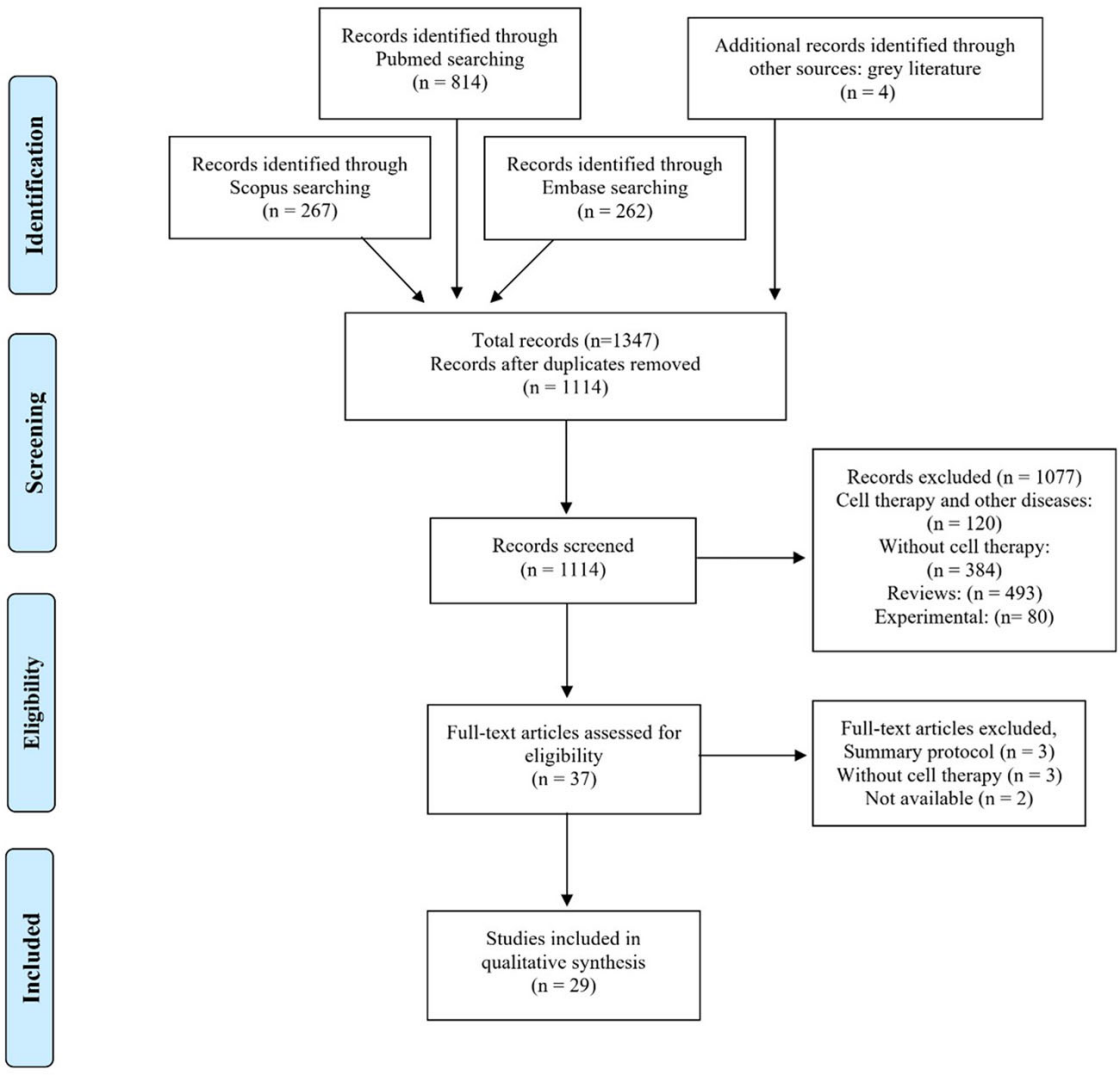
PRISMA flow diagram. Summary of evidence search and study selection.

The initial search of the databases identified a total of 1347 potentially relevant records. After excluding duplicates, 1114 articles remained, and titles and abstracts of the remaining records were scanned as part of a new screening according to inclusion and exclusion criteria previously established. Four gray literatures were also analyzed. A total of 1077 articles were excluded based on the exclusion criteria. The remaining 37 articles that met the inclusion criteria were thoroughly examined. Among these, eight were excluded, and the remaining 29 were included for the review. The PRISMA flow diagram (Fig. 1) illustrates the selection process of the studies for systematic review.

Among the clinical studies, 17 of them reportedly applied cell therapy to treat COVID-19, while the remaining eight studies investigated cell therapy as a treatment for ARDS. Additionally, four clinical COVID-19 articles were included as gray literature, bringing the total of selected clinical studies to 29. None of the included articles applied cell therapy to MERS or SARS-CoV-1.

The clinical and study characteristics are described in Table 1.

**Table 1:** Clinical and study characteristics.

Author	Disease	Cell type and administration	Number of patients	Inclusion criteria	Evaluated parameters	Main outcomes	Adverse effects	Study design/evidence level
Shi L.et al.^19^.	COVID-19	UC-MSC IV	100	- Severe COVID-19 - CT confirmed pneumonia and any of the following: (1) Noninvasive ventilation, shock, or other organ failures (2) RR ≥30 times/min; (3) O2 of 93% (4) PaO2/FiO2 ≤300 mmHg; (5) CXR/CT evidence of progression >50% in 24–48 h	Clinical outcomes Biomarker response inflammatory factors Leukocytes count	↓ Pro-inflammatory factors improvement on CT	Pneumothorax Not related	Phase 2 clinical trial: multicenter, double-blind, randomized, controlled. 1 A: high
Matthay MA. et al.^27^.	ARDS	BM-MSCs IV	60	(1) Endotracheally intubated (2) ↓ PaO2/FiO2 (3) Positive pressure ventilation (4) Bilateral infiltrates on CXR (5) No clinical evidence of left-heart failure or volume overload	Hospital indexes SOFA score Angiopoietin-2 Respiratory parameters Inflammatory factors Alveolar epithelial injury	↓ Angiopoietin-2 The results had no statistical relevance	Cardiopulmonary arrest Not related	Phase 2 clinical trial: multicenter, double-blind, randomized, controlled. 1 A: high
Zheng G. et al.^31^.	ARDS	AD-MSCs IV	12	ARDS diagnostic criteria according to the New Berlin definition	PaO2/FIO2 Hospital indexes Inflammatory factor biomarker responses	↑ PaO2/FIO2	Diarrhea and rash in chest area related	Clinical trial: single-center, randomized, double-blind, controlled. 1B: moderate
Lanzoni G. et al.^14^.	COVID-19	UC-MSC IV	24	(1) Hospitalized patient ≥18 years (2) SpO2 ≤94% (3) PaO2/FiO2 <300 mmHg (4) Bilateral infiltrates CXR or bilateral ground-glass opacities on a CT	Survival Inflammatory factors	↓ Pro-inflammatory factors ↑Survival/↓Mortality Improvement on hospital discharge ↓Biomarker responses	Bradycardia Related Acute respiratory failure not related	Phase 1 clinical trial: a single-center, double-blinded, randomized, controlled. 1B: moderate
Bellingan G. et al.^33^.	ARDS	BM-MAPC IV	30	(1) PaO2/FiO2 above or below 150 mmHg (2) Need for vasopressors (3) 96-h of moderate-to-severe ARDS onset	Clinical outcomes PaO2/FIO2 Hospital indexes Biomarker responses	↑ PaO2/FIO2 ↑Survival/↓Mortality Clinical improvement Improvement on hospital discharge Discontinuation of oxygen support ↓Biomarker responses	Single CTCAE grade 1 Related	Phase 1 clinical trial: multicenter, open-label, randomized, controlled. 1B: moderate
Meng F. et al.^17^.	COVID-19	UC-MSC IV	18	(1) Patients aged 18–70 years old. (2) Confirmed COVID-19 (3) Pneumonia evidenced by CXR or CT	inflammatory factor biomarker responses PaO2	↓ Pro-inflammatory factors ↑ PaO2/FIO2 Improvement on CT	Transient facial flushing Fever Related Hypoxemia Not related	Phase 1 clinical trial: a single-center, open-label, non- randomized, controlled. 2B: low
Shu L. et al.^18^.	COVID-19	UC-MSC IV	41	- PCR COVID-19 - CT indicators of pneumonia and any of the following: (1) respiratory distress, RR ≥30 times/min; (2) O2 saturation ≤93%; (3) PaO2/FiO2 ≤300 mmHg	Biomarker responses Inflammatory factors Leukocytes count	↓ Pro-inflammatory factors ↑ Lymphocyte count ↑ O2 saturation Improvement on CT Clinical improvement	None	Clinical trial: single-center open-label, randomized, controlled. 2B: low
Feng Y.et al.^21^.	COVID-19	UC-MSC IV	16	Severe COVID-19 using the Clinical classification by the National Health Commission of China	Biomarker responses Inflammatory factors Leukocytes count EKG CT	↓ Pro-inflammatory factors ↑ Lymphocyte count Clinical improvement ↑ PaO2/FIO2	Bacterial pneumonia Septic shock Not related	Pilot trial: multicenter, open-label, non- randomized, noncontrolled. 2B: low
Wilson J. et al.^26^.	ARDS	BM-MSCs IV	9	(1) Positive pressure ventilation ↓ PaO2/FiO2 (2) Bilateral infiltrates on CXR (3) No clinical evidence of left atrial hypertension (4) Criteria 1–3 must be present within a 24 h period and at the time of enrollment	Lung injury score (LIS) Angiopoietin-2 Alveolar epithelial injury Inflammatory factors SOFA score Biomarker responses	LIS improved ↓ SOFA The results had no statistical relevance	Respiratory arrest Sepsis Embolic infarcts Not related	Phase 1 clinical trial: multicenter, open-label, non- randomized, noncontrolled 2B: low
Sánchez-Guijo F. et al.^32^.	COVID-19	AD-MSCs IV	13	- PCR COVID-19 - Pneumonia diagnosed by CXR or CT requiring mechanical ventilation in the ICU and any of the following: (1) RR 30 times/min (2) finger O2 saturation 93% (3) PaO2/FiO2 <300 mmHg (4) CXR/CT evidence of progression >50% in 24–48 h (5) SOFA score >3 points (6) WHO OSCI level 6 (7) No evidence of multiorgan failure Compassionate use	Safety EKG CXR Biomarker responses Inflammatory factors Leukocytes count Clinical outcomes Hospital indexes	↓ Pro-inflammatory factors ↑ Lymphocyte count Improvement on CT/CXR Clinical improvement Discontinuation of oxygen support	Massive gastrointestinal bleeding Hypotension Tachycardia Pneumonia Fungal infection by Candida spp. Not related	Clinical trial: multicenter, open-label, non- randomized, noncontrolled. 2B: low
Leng Z. et al.^34^.	COVID-19	ACE2-MSCs IV	10	No improvement under the standard treatments	Biomarker responses Inflammatory factors Clinical outcomes Leukocytes count CT RR	↓ Pro-inflammatory factors ↑ Lymphocyte count ↑ O2 saturation Improvement on CT Clinical improvement	None	Pilot trial: a single-center, open-label, non- randomized, controlled. 2B: low
Tang L. et al.^36^.	COVID-19	Menstrual blood-derived SC IV	2	Not applicable	Hospital indexes Biomarker responses Inflammatory factors CT	↓ Pro-inflammatory factors ↑ Lymphocyte count Clinical improvement Improvement on CT	None	Pilot trial: multicenter, open-label, non- randomized, noncontrolled. 2B: low
Hashemian S. et al.^38^.	COVID-19	P-MSCs IV	11	(1) Patients with 18–70 years (2) Evidence of pneumonia in CXR or CT and/or COVID-19 positive by RT-PCR (3) ARDS diagnosed (4) SpO2/FiO2 ≤315 (5) SOFA score between 2 and 13 (6) Required mechanical ventilation and/or supplemental O2	Clinical outcomes Leukocytes count SOFA PaO2/FIO2 RR	↓ Pro-inflammatory factors Improvement on CT/CXR Clinical improvement ↓Biomarker responses	Transient shivering Related Cardiac arrest Not related	Phase 1 clinical trial: multicenter, open-label, non- randomized, noncontrolled 2B: low
Iglesias M. et al.^16^.	COVID-19	UC-MSC IV	5	(1) Severe ARDS -Berlin definition (2) PCR COVID-19 (3) No clinical improvement after standard management (4) Persistent fever (5) D-dimer by at least 50% up from the baseline value and/or ferritin concentrations >1000 ng/mL (6) CT with ground-glass opacity and bilateral pneumonia (7) SOFA <11 Compassionate use	Biomarker responses Inflammatory factors Leukocytes count CT RR, HR PaO2/FIO2	↓ Pro-inflammatory factors ↑ PaO2/FIO2 Improvement on CT ↑Survival/↓Mortality Discontinuation of oxygen support	Hypoxemia Hypotension and/or hypertension Muscle spasms Related Acute Kidney Injury Cardiomyopathy and Liver Failure Bacterial Pneumonia Bleeding Lower-extremity arterial thrombosis Epistaxis and hematuria Not related	Pilot trial: single-center, open-label, non- randomized, noncontrolled. 2 C: low
Singh S. et al.^22^.	COVID-19	CDC IV	6	(1) RT-PCR COVID-19; (2) severe COVID-19, requiring supplemental oxygen and/or shock requiring inotropes; (3) not enrolled in another clinical trial; Compassionate use	Inflammatory factors Leukocytes count Biomarker responses	↓ Pro-inflammatory factors ↑ Lymphocyte count ↑Survival/↓Mortality Clinical improvement Discontinuation of oxygen support Improvement on hospital discharge	None	Clinical trial: a single-center, open-label, non- randomized, noncontrolled. 2 C: low
Chen X. et al.^23^.	COVID-19	BM-MSCs IV	25	(1) Diagnosis of severe COVID-19; (2) age ≥18 years; (3) Receiving MSCs therapy	Clinical outcomes CT Leukocytes count Inflammatory factors Biomarker responses	Clinical improvement Improvement on CT ↑Survival/↓Mortality LAC, cTnT, and CK-MB elevated	Liver dysfunction Heart failure Allergic rash Related	Clinical trial: a single-center, open-label, non- randomized, noncontrolled. 2 C: low Retrospective
Helene H. et al.^28^.	COVID-19	BM-MSCs IV	23	(1) Confirmed COVID-19 (2) Horovitz index <100 on admission.	Biomarker responses Inflammatory factors Leukocytes count	↓ Pro-inflammatory factors ↓ Neutrophil count ↑ Lymphocyte count ↑ PaO2/FIO2 ↑Survival/↓Mortality Improvement on hospital discharge Discontinuation of oxygen support	None	Clinical trial: a single-center, open-label, non- randomized, noncontrolled. 2 C: low
Sengupta V. et al.^29^.	COVID-19	BM-MSCs IV	24	(1) RT-PCR COVID-19; (2) Fever and/or dyspnea for more than 72 h (3) ↓ PaO2/FiO2 ratio Compassionate use	PaO2/FIO2 Oxygen support requirements Inflammatory factors CXR; EKG Cultures	↓ Pro-inflammatory factors ↑ Lymphocyte count ↓ Neutrophil count ↑Survival/↓Mortality ↑ PaO2/FIO2 Clinical improvement Improvement on hospital discharge Discontinuation of oxygen support	Hypoxic respiratory failure Pulmonary embolism Acute renal failure Expiration Not related	Clinical trial: a single-center, open-label, non- randomized, noncontrolled. 2 C: low
Zhinian G. et al.^11^.	COVID-19	UC-MSC IV	31	Severe COVID-19 pneumonia	Inflammatory factors PaO2/FIO2 Leukocytes count MSCs regulation Biomarker responses	↓ Pro-inflammatory factors ↑ Lymphocyte count ↑ PaO2/FIO2	None	Clinical trial: a single-center, open-label noncontrolled, non- randomized. 2 C: low
Zhang Y. 1et al.^12^.	COVID-19	UC-MSC IV	1	Not applicable	Clinical outcomes Lung function Leukocytes count Inflammatory factors Biomarker responses	↓ Pro-inflammatory factors ↑ O2 saturation Improvement on CT Clinical improvement Improvement on hospital discharge	None	Case Report 4 C: very low
Chang Y. et al.^13^	ARDS	UC-MSC IT	1	Compassionate use	PaO2/FIO2 CT Mental status Lung compliance	↑ PaO2/FIO2 Clinical improvement Discontinuation of oxygen support His mental status, lung compliance, P/F ratio improved	Repeated pulmonary infections Septic shock empyema Not related	Case Report 4 C: very low
Liang B. et al.^15^.	COVID-19	UC-MSC IV	1	Not applicable	Leukocytes count Biomarker responses Inflammatory factors	↓ Pro-inflammatory factors ↑ Lymphocyte count ↓Biomarker responses ↓ Neutrophil count	None	Case Report 4 C: very low
Peng H. et al.^20^.	COVID-19	UC-MSC IV	1	No improvement under the standard treatments	Clinical outcomes Biomarker responses Inflammatory factors CT PaO2	↓ Pro-inflammatory factors ↓ Neutrophil count ↑ Lymphocyte count Clinical improvement Discontinuation of oxygen support Improvement on CT	None	Case Report 4 C: very low
Simonson O. et al.^24^.	ARDS	BM-MSCs IV	2	Two patients with severe ARDS treated on a compassionate use	Physical capacity HRQoL Lung function DECT	Improvement on CT Discontinuation of oxygen support Clinical improvement	None	Cohort: 5-year follow-up. 4 C: very low
Simonson O. et al.^25^.	ARDS	BM-MSCs IV	2	Refractory ARDS	BALF Inflammatory factors CXR and CT Clinical outcomes Respiratory measurements Biomarker responses	↓ Pro-inflammatory factors Discontinuation of oxygen support Improvement on CT Clinical improvement	Nosocomial pneumonia Not related	Case Series 4 C: very low
Jungebluth P. et al.^30^.	ARDS	PBMCs IT	1	Refractory ARDS	Gene expression Bronchoscopy Clinical outcomes Inflammatory factors	↓ Pro-inflammatory factors ↑ PaO2/FIO2 Improvement on CT Clinical improvement Discontinuation of oxygen support	Disseminated fungal infection Intra-abdominal sepsis Not related	Case Report 4 C: very low
Lu J. et al.^35^.	COVID-19	Menstrual blood-derived SC IV	1	No improvement under the standard treatments	CT Inflammatory factors	↓ Pro-inflammatory factors Improvement on CT	None	Case Report 4 C: very low
Wu J. et al.^37^.	COVID-19	IMRCs IV	1	No improvement under the standard treatments Compassionate use	Clinical outcomes CT Inflammatory factors Blood pressure, O2 saturation Leukocytes count	↓ Pro-inflammatory factors Clinical improvement	None	Case Report 4 C: very low
Tao J. et al.^63^.	COVID-19	UC-MSC IV	1	No improvement under the standard treatments Compassionate use	Blood gas analysis Clinical outcomes Biomarker responses	↓ Pro-inflammatory factors ↑ Lymphocyte count Clinical improvement ↓Biomarker responses ↑ PaO2/FIO2	Lung transplant rejection	Case Report 4 C: very low

All studies required informed consent. All studies availed safety. Hospital indexes: length of hospital stay, ventilator-free days and ICU-free days, length of time from admission to the start of mechanical ventilation, the time between the latter, and extubation or death.

*START* stem cells for ARDS treatment, *ATMP* advanced therapy medicinal product, *ACE2-MSC* angiotensin-converting enzyme 2 mesenchymal stem cells, *hESC* human embryonic stem cells, *UC-MSC* umbilical cord-derived mesenchymal stem cells, *CDC* cardiosphere-derived cells, *BM-MCSs* bone marrow-derived mesenchymal stem cells, *PBMCs* peripheral blood mononuclear cells, *AD-MSCs* adipose-derived mesenchymal stem cells, *BM-MAPC* bone marrow-derived multipotent adult progenitor cells, *IMRCs*- immunity and matrix-regulatory cells, *IV* intravenous, *P-MSC* perinatal tissues mesenchymal stem cells, *RT-PCR* real-time polymerase chain reaction, *PaO2/FiO2* arterial oxygen partial pressure/fractional inspired oxygen, *CRP* C-reactive protein, *ECMO* extracorporeal membrane oxygenation, *SOFA* *Score* sequential organ failure score, *CXR* chest X-ray, *CT* computed tomography, *ICU* intensive care unit, *RR* respiratory rate, *SpO2* oxygen saturation, *WHO OSCI* WHO-ordinal scale for clinical improvement, *DECT* dual-energy computed tomography, *BALF* bronchoalveolar lavage fluid, *EKG* electrocardiogram, *HR* heart rate.

### Study designs and evidence levels

Among the 29 included clinical studies, there were eight case reports (27.5%), five Phase I clinical trials (17.2%), four (13.7%) pilot studies, two Phase II clinical trials (6.8%), one cohort (3.4%), and one case series (3.4%). The study designs and classification of evidence are described in Table 2.

**Table 2:** Study designs and evidence levels.

Study design	Oxford evidence level	GRADE evidence level	Articles
Phase 2 clinical trial: multicenter, double-blind, randomized, controlled	1a	high	^19,27^
Clinical trial: single-center, randomized, double-blind, controlled	1b	moderate	^31^
Phase 1 clinical trial: multicenter, open-label, randomized, controlled	1b	moderate	^33^
Phase 1 clinical trial: single-center, double-blinded, randomized, controlled	1b	moderate	^14^
Phase 1 clinical trial: multicenter, open-label, non-randomized, noncontrolled	2b	low	^26,38^
Pilot trial: single-center, open-label, non-randomized, controlled	2b	low	^34^
Phase 1 clinical trial: single-center, open-label, non-randomized, controlled	2b	low	^17^
Clinical trial: multicenter, open-label, non-randomized, noncontrolled	2b	low	^32^
Clinical trial: single-center			
open-label, randomized, controlled	2b	low	^18^
Pilot trial: multicenter, open-label, non-randomized, noncontrolled	2b	low	^21,36^
Clinical trial: single-center, open-label, non-randomized, noncontrolled	2c	low	^11,22,23,28,29^
Pilot trial: single-center, open-label, non-randomized, noncontrolled	2c	low	^16^
Cohort	4c	very low	^24^
Case report	4c	very low	^13,20,24,30,35,37,63^
Case series	4c	very low	^25^

It should be noted that eight (27.5%) studies used cell therapy as compassionate use and one (3.4%) as a proof of concept. Most studies (75%) used mesenchymal stem cell therapy in an attempt to treat COVID-19.

The articles were categorized based on levels of scientific evidence following the Oxford Center for Evidence-Based Medicine Classification^9^ and the Grading of Recommendations, Assessment, Development, and Evaluations (GRADE)^10^.

According to the Oxford Center for Evidence-Based Medicine Classification^9^, of the 29 included articles, five were classified as grade A recommendations; within these, two (6.8%) were 1 A, and three (10.3%) were 1B. Among grade B recommendations, there were 14 studies. The levels of evidence were eight (27.5%) 2B and six (20.6%) 2 C. Among grade C recommendations, ten (34.4%) articles were classified as level 4 evidence.

According to the GRADE^10^, of the 29 included articles, two (6.8%) were considered high evidence level, three (10.3%) moderate, fourteen (48.2%) low, and ten (38.4%) minimal evidence level.

### Patient characteristics

Clinical studies using cell therapy for the treatment of COVID-19 patients were performed in five different countries. Following are the total numbers of patients by country from all 29 studies: China (*n* = 238), the United States (*n* = 30), Spain (*n* = 13), Iran (*n* = 11), and Germany (*n* = 23). Clinical studies that investigated ARDS included patients from the United States (*n* = 96), the United Kingdom (*n* = 30), China (*n* = 20), Sweden (*n* = 5), Mexico (*n* = 5), and South Korea (*n* = 1). Age of the patients ranged from 19 to 86 years.

### Intervention characteristics

Among the 29 selected clinical articles, eleven types of umbilical cord MSCs were analyzed. The cell types investigated in the studies comprised (1) human umbilical cord MSCs^11–21^, (2) cardiosphere-derived MSCs^22^, (3) human bone marrow MSCs (hBM-MSCs)^23–28^, (4) extracellular vesicles derived from BM-MSCs^29^, (5) autologous peripheral blood-derived mononuclear cells (PBMCs)^30^, (6) human adipose tissue-derived MSCs^31,32^, (7) allogeneic bone marrow-derived multipotent adult progenitor cells (MAPC) expanded ex vivo^33^, (8) ACE2-MSCs^34^, (9) human menstrual blood-derived MSCs^35,36^, (10) immunity‐and matrix-regulatory cells (IMRCs)^37^, and (11) MSCs derived from perinatal tissues^38^.

### Main parameters

A total of five groups of readouts—all of them subdivided into several parameters—were identified among the clinical studies. Laboratory measurements were common to 25 out of the 29 clinical articles, representing 86.2% of our study sample. The cited parameters included standard laboratory measurements, such as lymphocyte count, COVID-19 PCR test, basic metabolic panel (BMP) and levels of procalcitonin, ferritin, angiopoietin, d-dimer, bilirubin, alanine aminotransferase (ALT), creatinine, CKMB, cardiac troponin, and immune system parameters, such as IL-6, IL-8, IL-1ɑ, IL-1β, TNF-ɑ, and IL-1. Twenty studies, which correspond to 68.9% of the included studies, investigated pulmonary function using various parameters and exams, such as chest X-ray, bronchoscopy, chest CT, lung compliance, lung injury score (LIS), and the following biomarkers: receptor for advanced glycation end-product (AGER), which is a marker for lung epithelial injury, and angiopoietin-2 (ANGPT2), a marker for endothelial injury. All studies evaluated adverse reactions, safety, and mortality after the intervention. Ten studies (34.4%) analyzed the relationship between cell therapy and the discontinuation of ventilator support. Furthermore, eight studies (27.5%) documented additional data, such as mental status, patient’s physical capacity, health-related quality of life (HRQoL) assessment, e electrocardiogram (EKG), viral load, blood type and screen, blood culture, urine culture, and body mass index (BMI).

### Main outcomes

Among the 14 studies that evaluated lymphocyte counts before and after cell therapy, 12 reported a statistically significant elevation in lymphocyte numbers (85.7%). Fourteen of the seventeen studies which assessed CRP levels reported a decrease in this parameter (82.3%). As for plasma cytokines, cell therapy was found to reduce IL-6 levels in nine (52.9%) out of seventeen studies that investigated this parameter. TNF-α levels decreased in five out of seven studies (71.4%) that assessed its posttreatment values. Five out of six studies that measured ferritin reported a significant reduction after cell infusion, while six out of eight studies (75%) reported a D-dimer decrease. Remarkably, all studies that evaluated the following pulmonary parameters reported a statistically significant difference between pre- and post-cell therapy results: there was an increase in PaO2/FIO2 ratio in 12 out of 14 articles (85.7%), an increase in oxygen saturation in 50% of the articles and an improvement on lung image—chest CT or chest radiography—in 15 out of 17 studies (88.2%). Eighteen studies assessed symptoms at admission and clinical status after cell therapy, and all of them reported clinical improvement. Additionally, 11 out of 14 studies (78.5%) demonstrated discontinuation of oxygen support (intubation and ECMO) after cell infusion at a statistically significant rate. Nine studies reported a reduction in mortality, but two of them did not report a significant reduction (77.7%). Six studies evaluated and reported improvement in the duration of hospitalization. The primary outcomes and evaluated parameters are summarized in Table 3.

**Table 3:** Main outcomes and evaluated parameters.

Laboratory parameters	Studies
↑ Lymphocyte count	^11,15,19–22,28,29,32,34,36,63^
↓ C-reactive protein level	^11,12,16,17,19–22,29,30,32,34,36^
↓ IL-6	^11,12,14,17,19,21,22,35,36^
↓ TNF-α	^12,14,19,21,34^
↓ Ferritin	^17,22,28,29,32^
↓ D-dimer	^11,15,16,22,29,32^
Pulmonary parameters	Studies
↑ PaO2/FIO2 (Horowitz index) or P/F ratio	^11,13,16,17,20,21,28–31,33,63^
↑ Oxygen saturation	^12,19,34^
Improvement on chest CT/radiography	^12,16–20,23–25,30,32,34–36,38^
Other parameters	Studies
↑ Survival/↓Mortality	^14,16,22,23,28,29^
Clinical improvement	^12,13,18,20–22,24,25,29,30,32,34,36–38,63^
Improvement on hospital discharge	^12,14,22,28,29,33^
Discontinuation of oxygen support (intubation, ECMO)	^13,16,20,22,24,25,28–30,32,33^

### Evidence level: 1A and 1B studies

Studies in higher evidence level categories (1A and 1B) described some noteworthy findings. In a randomized phase 2 safety trial for patients with moderate-to-severe ARDS, Matthay et al. revealed a decrease in endothelial injury ascertained by reduced plasma concentrations of ANGPT2 in the MSC-treated patients versus the control group^27^. They also reported that after MSC therapy, there was a trend towards a decreased number of ventilator-free and organ failure-free days and improved oxygenation index, although not significantly^27^. Notably, the number of intensive care-free days was found to be statistically relevant. Additionally, they showed a reduction in the levels of CRP and ANGPT2 biomarkers^27^.

Shi and colleagues performed a multicenter, randomized placebo-controlled phase 2 efficacy trial with 100 severe COVID-19 patients, who either received placebo (*n* = 35) or umbilical cord mesenchymal stem cells (UC-MSC) infusion (*n* = 65) alongside the common care treatments. Supplementary oxygen was necessary for 44 (67.69%) patients from the treatment group and for 23 (65.71%) patients from the placebo group. They noticed that UC-MSC infusions could reduce the proportion of abnormal lung lesions, especially lesions with solid appearance, compared to placebo. In this study, MSCs led to a decrease in ground-glass lesions; however, it was not significant compared to the placebo group^19^. Both articles classified 1 A according to Oxford and GRADE systems described that cell therapy was a safe and well-tolerated alternative^19,27^. All three papers classified as 1B also concluded that the procedure was safe^14,31,33^. Zheng et al. in a single-center, randomized, double-blind, placebo-controlled trial in which 12 patients with moderate-to-severe ARDS were arbitrarily assigned to receive either allogeneic adipose-derived humans MSCs or placebo. They observed that the MSC group displayed a significant improvement in oxygenation index, compared to baseline values—but not compared to placebo. Parameters such as ventilator-free and ICU-free days and serum IL-6 and IL-8 levels did not show a difference when the baseline values were compared to predetermined time points^31^. Bellingan et al. reported that MSCs reduced mortality and increased ventilator-free and ICU-free days compared to placebo^33^. Lanzoni et al. conducted a controlled, double-blinded, randomized phase 1/2a clinical trial to determine the safety and efficacy of UC-MSC infusion in 24 patients with COVID-19 ARDS. The subjects were divided into a UC-MSC infusion (*n* = 12) and a placebo (*n* = 12) group. They noticed that UC-MSC infusion significantly contributed to improved patient survival and time of recovery. This study also reported a significant decrease in the following inflammatory cytokines after treatment: GM‐CSF (pro-inflammatory M1 macrophage phenotype inducer), IFN-γ, IL‐5, IL‐6, IL‐7, TNF-α, TNF- β, and others^14^.

### Evidence level 2B and 2C studies

Wilson and associates conducted a study to assess the safety and the maximally tolerated dose of MSCs - up to 10 million cells/kg predicted body weight (PBW). The study was a multicenter, open-label, non-randomized, noncontrolled phase 1 clinical trial, in which nine patients were equally subdivided into three intervention groups: low dose MSCs (1 million cells/kg PBW), an intermediate dose MSCs (5 million cells/kg PBW), and the high dose MSCs (10 million cells/kg PBW). After the treatment, the concentrations of several biomarkers (IL-6, IL-8, AGER, and ANGPT2) were decreased when compared between the baseline and day 3 values^31,33^. However, it is not possible to relate these findings to the previously reported changes in biomarkers, due to the lack of a matched control group with the MSCs treatment group in this study.

Hashemian and collaborators performed a multicenter, open-label, non-randomized, noncontrolled trial, with 11 subjects that either received freeze/thawed UC-MSCs (*n* = 6) or fresh PL-MSCs (*n* = 11). They noted that inflammatory biomarkers (CRP, IL-6, IL-8, and TNF-α) were significantly decreased after the MSC infusions. Notably, the anti-inflammatory cytokines IL-4 and IL-10 levels were also increased in four cases. Moreover, the study reported that nine (81.81%) treated patients tolerated the MSCs infusions^38^.

In a pilot study, Leng et al investigated the early efficacy of MSC therapy in seven patients with COVID-19 pneumonia along with the placebo treatment in three patients. Two days after infusions, they found improvements in pulmonary function, decreased levels of CRP and inflammatory cytokines, and augmented IL-10 concentration and regulatory DC cells^34^.

Meng F. et al. conducted a controlled, non-randomized, phase 1 clinical trial to evaluate the safety of human umbilical cord-derived MSC infusions in 18 patients. They observed that four patients displaying the highest levels of IL-6 showed a substantial decrease within 3 days of the treatment, but no such trend was observed in patients with low plasma IL-6. Regarding the PaO2/FiO2 ratio, there were improvements in most of the severe patients. Additionally, there was a decline in concentrations of the inflammatory cytokines TNF-α, IFN-γ, monocyte chemoattractant protein 1 (MCP-1), interferon-inducible cytokine IP-10 (IP-10), IL-22, interleukin 1 receptor type 1 (IL-1RA), IL18, IL-8, and macrophage inflammatory protein 1-alpha (MIP-1)^17^.

In a concept study, 13 severe COVID-19 patients requiring mechanical ventilation and receiving the standard care treatment were infused with adipose tissue-derived MSCs. Within 5 days of the treatment nine (70%) patients showed clinical improvements with significant reductions in CRP, lactate dehydrogenase (LDH), D-dimer, and ferritin. Moreover, five patients demonstrated improvements concerning B-lymphocyte alongside better CD4+ and CD8+ counts, and ultimately, after the MSCs infusion seven patients were extubated within a median time of 7 days^32^.

Shu, L. et al. conducted a single-center open-label, randomized trial involving 41 patients. Compared to the control group (*n* = 29), CRP and IL-6 levels were significantly decreased from day 3 after UC-MSC infusions (*n* = 12)^18^. In this study, the small sample size and the treatment change during the trial were the major limitations^18^.

Tang and colleagues tested the effects of infusions of menstrual blood-derived MSCs in two severe COVID-19 patients. They reported increased lymphocytes, decreased inflammation indicators (i.e., lower CRP and IL-6 levels) after MSC infusions, and antiviral treatment^36^.

Another pilot study conducted by Feng et al. investigated the efficacy of UC-MSCs in 16 patients; among them, seven were categorized as critically severe and nine as severe. They reported improvements in oxygenation index, CRP, and procalcitonin levels in severe and critically severe groups. In addition, the study noted augmented levels of CD4 + cells, CD8 + T cells, and NK cell counts within 28 days of the infusion^21^.

In a clinical trial involving MSC infusions in 31 patients, the PaO2/FiO2 level and lymphocyte counts showed a substantial increase, whereas the CRP, PCT, IL-6, and D-dimer were significantly decreased compared to the baseline values before infusions^11^.

In a single-center, open-label, non-randomized, noncontrolled conducted by Singh et al. in the earliest days of the COVID-19 outbreak, six patients received allogeneic cardiosphere-derived cells after receiving an anti-IL-6 agent. The levels of Ferritin, CRP, and IL-6 were decreased after the cardiosphere-derived cell infusion^22^. However, the decrease cannot be attributed only to cell therapy due to the prior treatment with anti-1L-6 agent^22^.

In a non-randomized open-label trial, Sengupta et al. apprised the safety and efficacy of an allogeneic bone marrow MSC-secreted extracellular vesicles (ExoFloTM) in 24 severe COVID-19 patients. Three days after infusions, they noted improvements in PaO2/FiO2 ratio (*p* < 0.001), significant increases in the absolute counts of neutrophils, CD3+, CD4+, and CD8+ lymphocytes, and decline in CRP, ferritin, and D-dimer levels after 5 days of ExoFloTM treatment^29^. The major limitations of this study were the lack of randomization and blinding.

Chen et al. performing a single-center, open-label, non-randomized, noncontrolled trial comprising 25 patients, reported improved CT parameters in 16 patients after MSC infusions (64%). However, there were no changes in inflammation indices, including CRP, WBC, PCT, and IL-6 levels. Moreover, there were no changes in IgG or IgM. In contrast, the serum levels of lactate, cardiac troponin T, and creatine Kinase were elevated after the treatment^23^. Low statistical power due to the small sample size was a significant limitation in this study.

Häberle and associates, in a single-center, open-label, non-randomized, noncontrolled trial, enrolled 23 patients to either placebo (*n* = 18) or MSC treatment (*n* = 5). No differences in CRP and IL-6 were found between the groups. Notably, ferritin level was increased in the MSC-treated group after discharge. Notwithstanding, there was a significant reduction in neutrophils, lymphocytes, and leukocytes at discharge in the MSC treatment group compared to the placebo group^28^.

### Evidence level 4C studies

Most studies with 4 C evidence levels were case reports, and hence the results could not be compared to findings seen in trials comprising larger cohorts of patients. It is also noteworthy that one cannot establish a cause–effect relationship from findings in case reports.

### Adverse effects

All clinical studies analyzed the occurrence of adverse effects (AE) related to cell therapy. Studies whose primary endpoint was safety mainly defined the occurrence of prespecified infusion-associated AEs within 6 h of infusions in addition to cardiac arrest or death within 24 h of infusions. A total of 16 studies (55.1%) reported the occurrence of side effects, with only a minority of the studies (24.1%) attributing the side effects directly to the use of cell therapy. Approximately 41% of the studies reported that the observed side effects were not linked to the MSC treatment. Twenty-seven (93.1%) studies did not report the AEs during the administration of MSCs. The AE are also detailed in Table 4.

**Table 4:** Adverse effects.

Adverse effects	Articles
AE related to mesenchymal cell therapy	^14,16,17,23,31,33,38^
AE not related to mesenchymal cell therapy	^13,16,17,19,21,25–27,29,30,32,38,63^
AE during the infusion	^16,17,31,33^
No AE	^11,12,15,18,20,22,24,28,34–37^
AE related to mesenchymal cell therapy	Articles
Worsening of Bradycardia	^14^
Hypoxemia Hypotension and/or hypertension Muscle spasms	^16^
Transient facial flushing Fever	^17^
Liver dysfunction Heart failure Allergic rash	^23^
Diarrhea Rash in the chest area	^31^
Single CTCAE grade 1	^33^
Transient shivering	^38^
AE not related to mesenchymal cell therapy	Articles
Repeated pulmonary infections Empyema	^13^
Lower-extremity arterial thrombosis Epistaxis and hematuria	^16^
Hypoxemia	^17^
Pneumothorax	^19^
Bacterial pneumonia Septic shock	^21^
Nosocomial pneumonia	^25^
Respiratory arrest Sepsis Embolic infarcts	^26^
Cardiopulmonary arrest	^27,38^
Hypoxic respiratory failure Pulmonary embolism Acute renal failure Expiration	^29^
Disseminated fungal infection and intra-abdominal sepsis	^30^
Massive gastrointestinal bleeding Hypotension Tachycardia Pneumonia Fungal infection by *Candida spp*.	^32^
Lung transplant rejection	^63^

### AE related to cell infusion

As for the side effects observed at the time of infusions or within hours after infusions, one study reported hypoxemia, hypotension and/or hypertension, and muscle spasms, but they were easily controlled and did not acutely alter the patients’ medical conditions^16^. A clinical trial observed transient facial flushing and fever immediately after the infusion, which resolved spontaneously^17^. One clinical trial detected diarrhea and a rash in the chest area, but it resolved^31^. Another article reported a single Common Terminology Criteria for Adverse Events (CTCAE) grade-1 infusion-related reaction, which settled without intervention^33^. One patient experienced transient shivering, which occurred once in two cases and disappeared in less than 1 h^38^.

### AE related to cell therapy

A study reported worsening of bradycardia in a subject who previously had bradycardia and required transient vasopressor treatment^14^. One pilot study observed liver dysfunction, heart failure, and allergic rash after treatment, but fortunately, all patients survived—the report did not specify the possible reason for the occurrence of these adverse reactions. Nevertheless, the serum levels of lactate, serum cardiac troponin, and creatine kinase were significantly increased after MSCs therapy, reinforcing the cautious use of MSC infusions in patients with previous metabolic acidosis and coronary heart disease^14^.

### AE not related to cell therapy

Adverse events unrelated to cell therapy included progressively increased creatinine, epistaxis, and hematuria; a patient was also diagnosed with lower-extremity arterial thrombosis^16^. Another patient experienced hypoxemia, which was thought to be caused by the progression of COVID-19 based on previously existing symptoms^17^. One patient experienced pneumothorax, which recovered spontaneously under conservative treatment, and it was judged by the site investigators and found to be unrelated to MSCs intervention^19^. Feng et al. reported two patients with bacterial pneumonia and septic shock as complications of severe COVID-19^21^. In a case series, one patient developed nosocomial pneumonia with fever several days after MSC infusions, but it was not related to cell therapy as per the report. Nonetheless, it remains to be addressed whether MSC infusions increase the risk of infectious complications in COVID-19 patients with ARDS, although no such increases were seen earlier in MSC clinical trials involving immune-competent recipients^25^. Wilson et al. reported multiple embolic infarcts, which were thought to be present before MSC infusions based on a previous MRI scan^26^. Another trial observed a worsening hypoxic state, a respiratory failure requiring intubation, pulmonary embolism, and acute renal failure. The reactions were reviewed by an independent Data Safety Monitoring Board (DSMB), which concluded that the symptoms were unrelated to the therapeutic intervention^29^. One subject experienced pneumonia due to a methicillin-resistant Staphylococcus aureus, and another patient developed a fungal infection by Candida spp^32^.

### Mortality

There were no reported deaths directly linked to stem cell administration. Death was observed in 17 studies, and a total of 79 patients died out of 472, including placebo and patients who received MSCs. It was reported that 47 (14%) deaths occurred among 330 patients receiving MSC infusions, while the placebo groups had 32 (23%) deaths out of 142 patients. Five studies described deaths caused by complications of COVID-19^11,16,18,21,29^. One patient experienced repeated infections by multidrug-resistant pathogens and eventually suffered septic shock and died^13^. A phase II trial reported two deaths in the MSC-treated group, secondary to acute respiratory failure; both were reviewed and declared as unrelated to MSC infusions^14^.

Iglesias et al. reported two deaths; the first patient developed acute renal failure and the second cardiomyopathy and liver failure^16^. A pilot trial observed two deaths caused by pneumonia and septic shock, which were lethal complications of COVID-19 and occurred independently of MSC treatment^21^. A phase I trial noted one death in the MSC treatment group and two deaths in the placebo group, but none of the deaths were considered to be related to MSC infusions by the clinical investigators and were consistent with the patients existing disease processes^26^.

One patient had a fatal cardiopulmonary arrest; however, it was attributed to a preexisting history of coronary artery disease, and the DSMB judged that the death was likely not related to MSC infusions^27^. In another clinical trial, one patient in the MSC infusions group died of multiple organ failure, which was concluded as not related to MSC infusions by the clinical investigators and was consistent with the patient’s disease progression^31^. Jungebluth et al. reported multisystem organ failure after MSC infusions, possibly secondary to disseminated fungal infection and intra-abdominal sepsis^30^. Among the two patients who died, one was due to gastrointestinal bleeding and another to secondary pneumonia, unassociated with MSC therapy^32^. Another trial reported five deaths after cell infusions, two were intubated, two had signs of sepsis, the fifth patient had a cardiac arrest, and none of the deaths were considered connected to cell therapy^38^. The mortality after MSC infusions is described in Table 5.

**Table 5:** Mortality.

Mortality MSC (death/*n*)	Mortality in control (death/*n*)	Reason of death	Articles
4/31	NA	Complications of COVID-19	^11^
1/1	NA	Septic shock after empyema	^13^
1/12	7/12	MSC: acute respiratory failure Placebo: multiple organ failure (6) and respiratory failure (1)	^14^
2/5	NA	Acute renal failure Cardiomyopathy and liver failure Bacterial pneumonia Complications of COVID-19	^16^
0/12	3/29	Complications of COVID-19	^18^
2/16	NA	Bacterial pneumonia and septic shock Complications of COVID-19	^21^
2/9	NA	Multiple organ failure Respiratory arrest	^26^
15/40	5/20	MSC: cardiopulmonary arrest (1) Multiple organ failure Not specified	^27^
1/5	10/18	MSC: multiple organ failure Placebo: acute liver dysfunction (4); multiorgan failure (3); palliative care (2); cerebral bleeding (1)	^28^
4/24	NA	Complications of COVID-19	^29^
1/1	NA	Multiple organ failure	^30^
1/6	2/6	MSC: multiple organ failure Placebo: multiple organ failure and sepsis	^31^
2/13	NA	Massive gastrointestinal bleeding due to a gastric ulcer Secondary fungal pneumonia	^32^
5/20	4/10	Not specified	^33^
0/7	1/3	Not specified	^34^
5/11	NA	Multiple organ failure Cardiac arrest	^38^
1/1	NA	Lung transplant rejection	^63^

## Discussion

The present systematic review evaluated the available results of stem cell therapy for treating patients with COVID-19 and ARDS. By July 2021, according to the US National Institutes of Health (NIH) ClinicalTrials.gov database, 417 clinical investigations of cell therapies against SARS-CoV-2 have been conducted^39^. The database search for this systematic review was conducted in March 2021. Following the inclusion criteria evaluation, 29 articles were eventually included. The vast number of trials under progress shows that cell therapy has been suggested as a beneficial alternative to treating COVID-19 and its complications ARDS. Among the 417 registered trials, 203 were recruiting participants, five were enrolling participants by invitation, 32 were active, having already passed the recruitment phase, and 63 were completed. Of these 63, six trials already presented results. The remaining 114 trials were not in progress or had an unknown status. Novel therapies for COVID-19 are being explored with the primary objective of ameliorating the rates of morbidity and mortality by reducing lung injury, improving host immunity, and decreasing inflammation. Previous clinical trials have suggested that cell therapy is safe and leads to multiple beneficial effects in diverse respiratory conditions^40–45^. Our evaluation of the literature also suggested similar findings for cell-based therapy to treat patients affected by COVID-19 and ARDS.

In a recent report, Kim and Knoepfler evaluated stem cell (including MSCs) and NK cell therapy results from two clinical trial registries comprising 79 cell therapy trials for COVID-19. Among these, 67.1% were randomized, 25.3% were double-blinded, 34.2% were placebo-controlled, and only 22.8% met all three criteria, namely randomization, double-blinding, and placebo controls^46^. In the present systematic review, we included 29 published articles in which six (20.7%) were randomized, four (13.8%) were double-blinded, eight (27.6%) were placebo-controlled, and four (13.8%) met all three criteria. Overall, the results of cell therapy trials for COVID-19 have not reached a high impact, as trials meeting the three primary criteria, randomization, double-blinding, and placebo controls have been minimal.

MSCs are extensively used in cell therapy since their efficacy and safety have been shown in several diseases in preclinical and clinical trials, with significant efficacy in inflammatory and immunologic conditions^47–52^. Most of the cell therapy studies reviewed here utilized MSCs as donor cells in patients with COVID-19 and ARDS. The most common sources of MSCs used to treat respiratory pathologies are bone marrow, umbilical cord blood, adipose tissue, and endothelial progenitor cells^53^. MSCs are characterized by their immunomodulatory functions combined with their regenerative and proliferative ability to help severely affected COVID-19 patients. These cells secrete a variety of paracrine factors that may facilitate immunomodulation and anti-inflammatory effects, which have been hypothesized as possible mechanisms contributing to improved lung function and regeneration in respiratory diseases^54,55^. As seen in most of the analyzed studies, the beneficial effects of MSC intervention in COVID-19 and ARDS appeared to be linked to improvements in the host immune system and inflammatory response. Such a conclusion is based on increased lymphocyte counts and decreased CRP and pro-inflammatory cytokine levels, facilitating lung repair.

Regarding the clinical outcomes analyzed in this study, three outcome domains were identified, which were further subdivided into several parameters: immune response, pulmonary function, and systemic response. Although most cases of COVID-19 are mild to moderate in severity, ~15% of cases evolve to severe pneumonia, and ~5% progress to ARDS and multiple organ failure. The progression to a worse presentation is related chiefly to cytokine upregulation and exaggerated inflammatory response. In severe ailing patients, a hyperimmune reaction termed cytokine storm results in critical illness and end-organ dysfunction, with a high mortality rate^22^. Findings from the included articles indicate a possible positive impact of cell therapy on crucial immunological and inflammatory processes that lead to organ injury in SARS-CoV-2-infected patients. Laboratory measurements were common to 25 of the 29 included clinical articles, representing 86.2% of published reports. Among the clinical laboratory findings, 18 studies demonstrated improvements in immunomodulatory responses or inflammation markers. Therefore, reversing or even attenuating the cytokine storm appeared to be a lifesaver in patients with severe COVID-19^11^.

The primary site of SARS-CoV-2 infection is the respiratory system. The virus is responsible for injuring the lungs and causing impaired alveolar oxygenation, hypoxemia, and acidosis. Dysregulated pulmonary functions following SARS-CoV-2 infection should be considered, as it can progress to death or permanent lung injury if the patient recovers^56^. Therefore, notwithstanding the miscellaneous nature of measures exploited in different studies, 23 studies (79%) evaluated pulmonary functions. All these studies evaluated pulmonary functions using a variety of parameters, such as PaO2/FIO2, oxygen saturation ratio, lung imaging—chest CT or chest radiography, symptoms at admission and clinical status after cell therapy, discontinuation of oxygen support (intubation and ECMO) after cell infusion and reduction in mortality. In these studies, noteworthy improvements were reported between pre- and post-cell therapy status.

The studies categorized as evidence levels 1A and 1B reported that clinical outcomes could be influenced by cell preparation techniques leading to considerable differences in MSC viability^27^. Bellingan and colleagues also reported increased intensive care-free days, as well as a reduction in mortality^33^. Lanzoni and associates showed a considerable reduction in the concentration of pro-inflammatory factors. However, the small sample size and modified inclusion criteria during the trial are some of the limitations in these studies^14^.

Several studies with evidence level B demonstrated a decrease in pro-inflammatory biomarkers after cell therapy intervention^11,17,18,22,29,34,36,38^, improved lung function^34^, reduced B-lymphocyte counts^11,21,28,29,32^ and better oxygenation levels^11,16,17,21,23,31,33,34^. However, these studies have significant limitations, including the lack of details on MSC origin or isolation^34^, the small sample size^18,21,32,38^, variability in MSC doses administered to patients^31,33^, irregular regimens of MSC administration^32^, insufficient cells for all the randomized patients assigned to receive cell infusions resulting in their assignment to the control group^18^, lack of a control group^11,21,23^, variability of treatments the patients received prior to cell infusions^22^, and the study design classified as retrospective^23^. Regarding 4 C evidence level studies, most of them were case reports, and hence, the extrapolation of these results to larger cohorts of patients will be difficult^24^.

Steroids act through similar proposed immune mechanisms as MSCs, the predominant cell type employed for infusions in the evaluated articles. Corticosteroid treatment for COVID-19 has proven to reduce mortality of patients on respiratory support^57^. However, some studies have discussed a few possible AE caused by the prolonged use of steroids, including hypertension, fluid retention, and increased risk of infection^58^. In addition, critically ill patients can have signs of hypercortisolism due to suppression of the neuroendocrine system, leading to the adrenal deficiency that could enhance mortality risk^59^, but it would differ based on individual variability in pharmacokinetics. The studies performing stem cell intervention reported restoration of lung function possibly through immunomodulation with just two transfusions, raising the debate that cell therapy could be beneficial in cases where patients do not respond well to standard treatment with steroids due to individual variability.

When proposing new therapeutic approaches, mortality is an important consideration, and this was one of the principal outcomes we reviewed in the present study. As per the reports, there were no deaths directly linked to cell therapy. Deaths were reported in 17 studies, and a total of 79 patients died. In all cases, it was suggested by the authors and committees that none were related to MSC infusions, but few studies were unsure about the cause of death. The studies also suggested that adverse events following MSC infusions in COVID-19 patients are rare or milder when present. The reported adverse events included facial flushing, transient fever, and hypoxia, but these were primarily attributed to the progression of COVID-19 and not directly to cell infusions^17^. Furthermore, cardiac arrest was reported 2 h after the infusion of the vehicle or cells in control and treated groups. These events were attributed to the infusion protocol (catheterization) rather than the MSC infusions per se^14^. Additionally, a study using MSCs for the treatment of ARDS reported a decrease in the mortality rate when patients received the therapy within 28 days. However, after 60 days, 38% of patients died. Still, the researchers concluded that the treatment was able to moderate the severe form of the disease and that the mortality in the treated group was not statistically higher than in the control group. They found that patients who received treatment with MSCs had numerically higher disease severity scores before the proposed treatment^27^. Besides, some of the collateral effects, including increased levels of lactate, serum cardiac troponin, and creatine kinase after MSC infusions, which resulted in liver dysfunction and heart failure, need to be investigated to determine their possible correlation with cell therapy and caution for patients with metabolic acidosis or coronary heart disease^23^. One study addressed the need for studies that correlate MSCs with an increased risk of infections; however, no such increase was reported in clinical trials^25^. All AE were controlled, indicating that cell therapy for treating COVID-19 and ARDS is likely safe.

Since the emergency pandemic situation broke out only recently, there was not enough time to produce a larger number of clinical trials with higher evidence levels, enrolling larger cohorts of patients and randomized controlled and multicenter studies involving cell-based therapy in severely affected COVID-19 patients. Randomized controlled trials have been pivotal to testing the safety and efficacy of a variety of interventions^60^. The novelty regarding the appraisal of safety and efficacy of stem cell infusions as an adjuvant treatment for COVID-19 is another critical issue that possibly explains the large number of low evidence studies discussed in this review.

In particular, we highlighted the efficacy of MSC therapy to halt the cytokine storm caused by SARS-CoV-2 infection, evident from the downregulated expression of pro-inflammatory cytokines and an increased concentration of an anti-inflammatory cytokine. Recently, COVID-19 was identified as a multi-organ infection with many symptoms linked to persistent manifestations and long-term effects on the cardiovascular and nervous systems after acute COVID-19^61^. In this context, MSC therapy could also help treat the long-term effects of COVID-19 infection, especially those emerging from chronic inflammation.

The number of patients recruited for the clinical studies was low since some trials were explored as a proof of concept and employed as compassionate use. Because the SARS-CoV-2 outbreak is still recent, there has not been enough time to produce extensive randomized clinical trials with a larger number of patients to understand better the effects of stem cell therapy on COVID-19-induced ARDS. Additionally, some of the analyzed studies are still in initial phases, and hence, our evaluation of findings was on the preliminary outcomes. Likewise, some studies did not use a control group, making the evaluation of the efficacy of MSC infusions difficult. Moreover, studies changed the course of treatment during the trial due to the lack of MSCs. Additionally, causes of death were unclear in some cases and were considered as unrelated to MSC infusions in others without adequate analysis and discussion on the issue. Therefore, additional clarifications are needed on how the cause of death was inferred as unrelated to cell infusions.

Furthermore, several aspects of cell therapy have not been satisfactorily elucidated. These include the best source of MSCs, the optimal dose of MSCs, the frequency of IV administration, and the therapeutic window for cell therapy intervention after COVID-19 infection to provide maximal protection to the lungs and other organs. It is also essential to consider that, according to Oxford and GRADE evaluation, most studies included here did not meet the highest level of evidence (A), and most of them were characterized as very low to moderate levels. Furthermore, it is too early to speculate on the exact mechanism by which stem cell therapy improves pulmonary complications, although this systematic review strived to provide provocative discussions.

Increased mortality worldwide due to SARS-CoV-2 infection and the emergence of new variants of the coronavirus emphasize the need to develop new therapeutic strategies for treating complications of COVID-19 and associated respiratory diseases such as ARDS. Treating COVID-19 complications is challenging since no treatment regimen has been entirely successful until now. Hence, identifying an effective and safe therapy for patients with severe COVID-19 is crucial for saving lives.

The use of cell therapy, especially MSCs, to treat COVID-19 appears promising based on the observations and findings in published studies. MSC therapy has shown promise to suppress cytokine storms, prevent the overactivation of the immune system, and repair the lung injury caused by SARS-CoV-2 infection. While the mechanisms of action of these cells are not yet fully understood, the beneficial effects promoted by MSCs are noteworthy. Thus, considering the current global pandemic situation, cell-based therapy could be considered an alternative treatment to containing the public health crisis, such as outbreaks in hospitals and care units and the collapse of medical infrastructure. Additionally, vaccines are already reducing the overall COVID-19 cases in many countries. However, cell-based therapy could also be used to treat the long-term sequelae caused by SARS-CoV-2 infection in patients, especially those related to chronic inflammation. For example, one in eight survivors of COVID-19 is diagnosed with neurological and psychiatric conditions, which may comprise confusion, problems in concentrating and memory recall, or depression. Nevertheless, more extensive double-blind, placebo-controlled, and multicenter clinical trials with a larger cohort of patients are necessary to validate the safety and efficacy of this therapeutic strategy.

## Methods

This review was conducted in accordance with the Preferred Reporting Items for Systematic Reviews and Meta-Analyses (PRISMA) Statement^62^ and was registered at the International Register of Prospective Systematic Reviews under identification number CRD42021248263.

### Search strategy

A systematic search was conducted in MEDLINE (via PubMed), Embase, and Scopus. Comparable elements of research were used for all databases with some adaptations to each database search system. The descriptors comprised “COVID-19”, “SARS-CoV-2”, “Coronavirus”, “2019-nCoV”, “SARS”, “MERS”, “acute respiratory distress syndrome”, and “cell therapy.” Data were obtained from published articles from January 2000 to March 2021. There were no language restrictions.

### Study selection

Three authors (L.L.L., L.P., and S.C.B.) independently assessed potentially eligible studies for their suitability for inclusion in this review. Disagreements were resolved by a fourth reviewer (GZ). During the screening of titles and abstracts, relevant papers were defined if they mentioned aspects of cell therapy applied as a therapeutic possibility for patients with COVID-19, ARDS, SARS, or MERS. Abstracts were analyzed according to the inclusion criteria, and all studies that met these criteria were included for full article reading.

Studies were included if they pertained to any stem cell therapy applied as a therapeutic alternative for patients with COVID-19, ARDS, SARS, or MERS with original data. The exclusion criteria were (1) review articles (including critical letters and systematic reviews), (2) studies with no mention of cell therapy, (3) studies relating to cell therapy applied to unspecified respiratory diseases, and (4) experimental studies. Figure 1 displays a flowchart of study selection and inclusion.

### Data extraction

Regarding the appraisal of the data from all included studies, five tables were structured to summarize each study’s characteristics and findings, main parameters and outcomes, study design and classification of evidence, AE, and mortality. Table 1 shows clinical studies detailing the title, authors, investigated disease, donor cell type, route of cell administration, participant characteristics, *n* of the study, inclusion criteria, analyzed parameters, and primary outcomes. Table 2 summarizes the study design and classification of evidence from each study assessed using the Oxford Centre for Evidence-Based Medicine and the Grading of Recommendation Assessment Development and Education (GRADE) systems. Table 3 summarizes the key evaluated parameters separated into three domains: laboratory, pulmonary, and other parameters. Table 4 summarizes the AE encountered in different studies. Table 5 summarizes the mortality data described in different studies.

## Data Availability

The data are available from the corresponding author upon reasonable request.
